# Therapeutic Advances in Metastatic Prostate Cancer: A Journey from Standard of Care to New Emerging Treatment

**DOI:** 10.3390/ijms262311665

**Published:** 2025-12-02

**Authors:** Rossella Cicchetti, Martina Basconi, Giulio Litterio, Angelo Orsini, Marco Mascitti, Alessio Digiacomo, Gaetano Salzano, Octavian Sabin Tătaru, Matteo Ferro, Carlo Giulioni, Angelo Cafarelli, Luigi Schips, Michele Marchioni

**Affiliations:** 1 Department of Medical Oral and Biotechnological Science, Università degli Studi “G. d’Annunzio” of Chieti, 66100 Chieti, Italy; giulio.litterio@gmail.com (G.L.); orsini.ang@gmail.com (A.O.); mar.mascitti@gmail.com (M.M.); digiacomoale90@gmail.com (A.D.); gaesalza@gmail.com (G.S.); luigi.schips@unich.it (L.S.); mic.marchioni@gmail.com (M.M.); 2 Department of Anatomy, Surgery and Anesthesiology and Intensive Care, Faculty of Medicine, University Dimitrie Cantemir, 540545 Targu Mures, Romania; sabin.tataru@gmail.com; 3 Department of Urology, ASSR Santi Paolo e Carlo, University of Milan, 20122 Milan, Italy; matteo.ferro@unimi.it; 4 Casa di Cura Villa Igea, 60127 Ancona, Italy; carlo.giulioni9@gmail.com (C.G.); info@angelocafarelli.it (A.C.)

**Keywords:** metastatic prostate cancer, abiraterone acetato, enzalutamide, apalutamide, chemotherapy in prostate cancer, radiopharmaceuticals, target therapy, PARP-inhibitor, PI3K/AKT/mTOR inhibitors, immunotherapy

## Abstract

Prostate cancer (PCa) remains one of the most prevalent malignancies among men worldwide and continues to pose significant therapeutic challenges, especially in its metastatic and castration-resistant forms. Over the past two decades, the treatment paradigm has evolved from monotherapy with androgen deprivation therapy (ADT) to a multifaceted approach integrating chemotherapy, androgen receptor axis-targeted therapies (ARATs), radiopharmaceuticals, and precision medicine. This review explores the molecular underpinnings of PCa, including genetic and epigenetic alterations such as BRCA1/2, TP53, and PTEN mutations, and their role in disease progression and treatment resistance. We detail the evidence supporting the integration of systemic agents like abiraterone, enzalutamide, and darolutamide into both hormone-sensitive and castration-resistant settings. Furthermore, we highlight the expanding role of radioligand therapies, including radium-223 and Lutetium-177-labeled PSMA-617 (Lu-PSMA-617), as well as the growing impact of PARP inhibitors in genomically selected patients. The emergence of theranostic strategies and next-generation sequencing has paved the way for personalized treatment algorithms, moving toward a truly precision oncology model in PCa. This comprehensive review synthesizes current therapeutic strategies, clinical trial evidence, and future directions aimed at optimizing outcomes and quality of life for patients with advanced prostate cancer.

## 1. Introduction

Prostate cancer (PCa) is the most common cancer in men in the U.S., with ~288,000 new cases in 2023, and its global incidence is expected to nearly double by 2040 [1,2]. While localized PCa has a favorable prognosis (5-year survival > 90%), advanced disease carries poor outcomes, with 5% presenting with metastases and 30–40% of treated patients experiencing biochemical recurrence [3,4,5].

Metastatic PCa is classified by response to androgen deprivation therapy (ADT) into hormone-sensitive (mHSPC) and castration-resistant (mCRPC) disease, the latter progressing despite castrate testosterone levels (<50 ng/dL) [6,7]. Progression to mCRPC is defined by biochemical evidence and then the rise of prostate-specific antigens (PSAs) or radiological evidence of disease advancement despite ongoing ADT [8].

First-line treatment for mHSPC typically includes ADT combined with additional systemic therapies such as docetaxel chemotherapy or androgen receptor-targeted agents (ARATs) including abiraterone, enzalutamide, apalutamide, and darolutamide. In mCRPC, treatment options include second-generation ARATs, chemotherapy (docetaxel or cabazitaxel), and molecularly targeted therapies [9].

This review summarizes current strategies and emerging therapies for metastatic PCa to guide optimal patient management.

## 2. Metastatic Prostate Cancer

ADT remains a cornerstone in the management of mPCa [8]. ADT refers to a therapeutic approach designed to suppress androgen production by interfering with the hypothalamic–pituitary–gonadal axis; this definition includes both LHRH agonists and antagonists. Chronic administration of LHRH agonists leads to desensitization and subsequent downregulation of pituitary LHRH receptors, resulting in the suppression of luteinizing hormone (LH) and follicle-stimulating hormone (FSH) secretion [8]. This hormonal suppression effectively reduces testicular testosterone production. Castrate levels of serum testosterone are typically achieved within 2 to 4 weeks following initiation of therapy [10]. At the start of ADT offer LHRH antagonists or orchiectomy to patients with impending clinical complications such as spinal cord compression or bladder outlet obstruction [11].

ADT with treatment intensification is strongly recommended for patients with mHSPC. While it demonstrates clear efficacy across disease stages, the most substantial benefits in terms of quality of life are observed in patients with advanced or metastatic disease, especially when used in combination with radiotherapy (RT) [12]. In the setting of newly diagnosed mHSPC, ADT alone provides a median overall survival (OS) of approximately 50 months [12]. The use of ADT monotherapy in this setting is discouraged unless there are clear contraindications to combination therapy. Treatment intensification options include doublet therapy of ADT with abiraterone, apalutamide, or enzalutamide; triplet therapy of ADT with docetaxel and abiraterone or darolutamide; or ADT with external beam radiation therapy (EBRT) to the primary tumor for low-metastatic burden [13].

Most advanced disease eventually stops responding to traditional ADT and is categorized as castration-resistant (also known as castration-recurrent) [14]. For patients who develop CRPC, ADT with an LHRH agonist or antagonist should be continued to maintain castrate serum levels of testosterone (<50 ng/dL). Patients with CRPC and no signs of distant metastasis on conventional imaging studies (M0) can consider monitoring with continued ADT if PSA doubling time (PSADT) is greater than 10 months (preferred), because these patients will have a relatively indolent disease history [15]. Secondary hormone therapy with continued ADT is an option mainly for patients with shorter PSADT (≤10 months) as described subsequently [16] ADT is continued in patients with mCRPC while additional therapies, including secondary hormone therapies, chemotherapies, immunotherapies, radiopharmaceuticals, and/or targeted therapies, are sequentially applied, as discussed in the subsequent sections; these findings underscore the importance of appropriately selecting patients and optimizing treatment combinations to enhance both survival outcomes and quality of life [17].

### 2.1. Use of ADT in Metastic Prostate Cancer

ADT was initially employed in the treatment of metastatic PCa as intermittent therapy. However, with the emergence of three pivotal phase III randomized trials (CHAARTED, STAMPEDE, and LATITUDE), the use of intermittent ADT has been superseded by continuous ADT-based combination therapy [18]. Initiation of therapy prior to the development of clinical symptoms is advised for most patients with mHSPC. A 2019 Cochrane review on this topic concluded that early administration of ADT likely prolongs OS as well as disease-specific survival [19].

The CHAARTED (Chemo-hormonal Therapy versus Androgen Ablation Randomized Trial for Extensive Disease in Prostate Cancer) trial introduced a clinically relevant stratification of patients with metastatic PCa based on disease burden, distinguishing between high-volume and low-volume disease [20]. High-volume disease was defined as the presence of either visceral metastases or ≥4 bone lesions, with at least one located outside the axial skeleton (i.e., beyond the spine and pelvis). Conversely, patients not meeting these criteria were classified as having low-volume disease [21,22]. The randomized phase III CHAARTED trial evaluated combination therapy with the addition of docetaxel to ADT in patients with mPCa, with OS as the primary endpoint [23]. The results demonstrated an improvement in OS in patients with high-volume disease, with a median survival of 51.2 months in the combination therapy group compared to 34.4 months in the ADT-only group [20]. The study concluded that docetaxel in combination with ADT should be considered a standard of care in patients with high-volume metastatic disease [22].

In the GETUG-15 trial, all enrolled patients had M1 stage PCa, either de novo or following prior local therapy. Stratification was performed based on previous treatments and Glass risk factors [21]. Patients received a combination of ADT and up to nine cycles of docetaxel. Similar to findings from previous studies, the addition of docetaxel resulted in an improvement in OS, particularly among patients with high-volume metastatic disease [21].

The multi-arm, multi-stage STAMPEDE trial investigated the efficacy of combining ADT with additional agents (e.g., docetaxel, abiraterone) compared to standard ADT monotherapy [24]. No stratification was applied based on metastatic disease volume. The findings regarding the addition of docetaxel to ADT were consistent with those observed in the CHAARTED trial [25]. Furthermore, the combination of abiraterone and ADT demonstrated a significant improvement in both OS and progression-free survival (PFS), including in patients with low-volume metastatic disease [26].

### 2.2. Use of Androgen-Receptors Axis Targeted Therapies (ARATs) in Metastatic Prostate Cancer

The conventional treatment approach for mHSPC has historically involved ADT alone. However, multiple clinical trials have shown that adding chemotherapy and/or ARATs to ADT leads to improved oncologic outcomes [27]. The progression and proliferation of CRPC are associated with elevated intratumoral androgen concentrations, primarily due to the upregulation of enzymes involved in androgen biosynthesis [28]. Furthermore, overexpression of the androgen receptor (AR), along with the emergence of mutated AR variants, contributes to tumor advancement [29]. ARATs such as abiraterone acetate and enzalutamide, represent a class of agents approved for the management of mCRPC, each acting through distinct molecular mechanisms [30].

#### 2.2.1. Abiraterone Acetate

Abiraterone acetate (COU-AA-302, NCT00887198) is an orally administered, irreversible inhibitor of the cytochrome P450 enzyme CYP17A1, which possesses dual 17α-hydroxylase and 17,20-lyase activity and plays a pivotal role in the steroidogenic pathway of androgen synthesis [29]. Inhibition of CYP17A1 disrupts the conversion of 17-hydroxypregnenolone into dehydroepiandrosterone (DHEA), thereby decreasing circulating concentrations of testosterone and other androgenic steroids [31]. This enzymatic blockade also leads to the accumulation of upstream mineralocorticoid precursors, such as 11-deoxycorticosterone, which can induce secondary hyperaldosteronism [32]. Consequently, concurrent administration of corticosteroids (prednisone) is required to mitigate this endocrine imbalance [33].

The addition of abiraterone acetate and prednisone (APP) to ADT has demonstrated a significant improvement in OS in men with mHSPC, as shown in one of the treatment arms of the STAMPEDE trial [34,35]. A similar benefit was observed in the LATITUDE trial, which specifically enrolled high-risk, de novo metastatic patients, reporting an OS hazard ratio of 0.62 (95% CI: 0.51–0.76), closely aligned with the STAMPEDE results [HR 0.63 (95% CI: 0.52–0.76)] [26,36]. Although the LATITUDE trial was restricted to high-risk patients, a post-hoc analysis of the STAMPEDE data revealed a comparable survival benefit irrespective of baseline risk classification or metastatic burden [37]. A similar improvement was observed in PFS, defined as the time to radiologic disease progression (median 16.5 vs. 8.2 months, HR: 0.52, *p* < 0.001) [38]. No significant difference was found in overall mortality rates between patients treated ADT alone and those receiving combination therapy with abiraterone acetate plus prednisone (AAP) [39]. In evaluating the safety profile of AAP in combination with ADT particular attention was given to the incidence and severity of treatment-related adverse events. The addition of AAP did not result in an increased incidence of grade III–IV musculoskeletal, gastrointestinal, respiratory, or general disorders [36]. However, there was an approximately threefold increase in the risk of acute cardiac toxicity (Peto OR = 2.93; 95% CI: 1.74–4.93; *p* < 0.001) and hepatic toxicity (Peto OR = 3.09; 95% CI: 2.12–4.50; *p* < 0.001), as well as a near twofold increase in grade III–IV vascular adverse events (OR = 2.28; 95% CI: 1.71–3.03; *p* < 0.001), the vast majority (≥90%) of which were attributable to hypertension [39,40]. However, toxicity profiles differed between the STAMPEDE (20%) and LATITUDE (10%) trials (see Table 1). The combination of AAP with ADT may be considered as a first-line treatment option in de novo metastatic patients who are deemed fit for systemic therapy [41].

According to the phase 3, multicenter, randomized PEACE-1 trial, the efficacy of triplet combination therapy was evaluated in terms of OS and radiographic progression-free survival in patients with de novo metastatic castration-sensitive (mCSPC) [42]. Between 2013 and 2018, enrolled participants received ADT combined with docetaxel, in addition to either abiraterone alone, abiraterone plus prednisone, or radiotherapy [43]. The majority of patients had low-volume disease. The findings demonstrated a significant improvement in clinical outcomes, with an increase in OS reaching 4.4 years and radiographic progression-free survival extending to 3.5 years in patients treated with the triplet regimen of ADT, docetaxel, and abiraterone [43]. However, this therapeutic approach was associated with increased treatment-related toxicity, most notably hypertension [44]. An updated analysis of the PEACE-1 trial, published in 2024, evaluated patients with mCSPC treated with a combination of ADT, docetaxel, and abiraterone, in conjunction with radiotherapy [43]. The results demonstrated a marked improvement in radiographic progression-free survival, while the benefit in OS was less pronounced [45]. With a median follow-up of 6.0 years (IQR 5.1–7.0), the addition of prostate radiotherapy did not result in a statistically significant improvement in OS among patients with low-volume disease (median OS 7.5 vs. 6.9 years; HR 0.98; 95.1% CI 0.74–1.28; *p* = 0.86), even when combined with abiraterone. These findings suggest that, although radiotherapy delays disease progression and castration resistance, it does not translate into a survival advantage in this setting [46]. Notably, the addition of radiotherapy was associated with a reduction in genitourinary adverse events and overall treatment-related toxicity [47]. These findings support the potential integration of radiotherapy into the standard therapeutic approach for mCSPC, regardless of metastatic volume [46].

#### 2.2.2. Enzalutamide, Apalutamide, Darolutamide

Next-generation non-steroidal antiandrogens exhibit enhanced affinity for the androgen receptor (AR) and, unlike earlier agents, inhibit AR nuclear translocation, eliminating partial agonist activity [48,49,50,51]. The main therapeutic lines involving these agents are schematically summarized in Table 1.

Enzalutamide, an oral AR signaling inhibitor, acts at multiple levels by blocking androgen binding, nuclear translocation, and DNA interaction [52]. Darolutamide, structurally distinct, demonstrates limited blood–brain barrier penetration in preclinical models, potentially reducing central nervous system-related adverse effects [53].

The phase III ARCHES study investigated the therapeutic benefit and safety profile of combining enzalutamide ADT in patients with mHSPC [54]. A total of 1150 patients were randomly assigned in a 1:1 ratio to receive either enzalutamide (160 mg daily) or placebo, both alongside ADT, with randomization stratified by disease burden and prior docetaxel treatment [55]. The primary outcome, radiographic progression-free survival (rPFS), was significantly prolonged in the enzalutamide arm (HR 0.39; 95% CI, 0.30–0.50; *p* < 0.001), with median rPFS not reached compared to 19.0 months in the control group [56]. Enzalutamide also led to significant reductions in the risks of PSA progression, initiation of subsequent anticancer therapies, symptomatic skeletal complications, development of castration resistance, and pain progression [57]. The incidence of grade 3 or higher adverse events was similar between groups (24.3% vs. 25.6%), and no unexpected toxicities emerged [58].

In the phase III ENZAMET trial, OS was the primary endpoint. At the time of the initial analysis, 102 deaths were reported in the enzalutamide plus ADT group compared to 143 in the standard-of-care group (ADT plus a non-steroidal antiandrogen like bicalutamide, nilutamide, or flutamide), resulting in a significant improvement in OS with enzalutamide (HR = 0.67; 95% CI: 0.52–0.86) [59]. Concurrent docetaxel was administered in approximately 50% of patients, 40% had received prior local therapy, and nearly half of the cohort presented with low-volume metastatic disease [60]. In a planned long-term analysis, with a median follow-up of 68 months, the survival benefit of enzalutamide was maintained (HR = 0.70; 95% CI: 0.58–0.84) [61]. Enzalutamide, when added to ADT in patients with mHSPC, is associated with delayed onset of pain deterioration and preservation of emotional well-being, contributing to improved overall health-related quality of life compared to ADT alone or in combination with older therapies (ADT alone, ADT with first generation nonsteroideal anti-androgens or ADT with docetaxel) [62]. In the phase III, double-blind, randomized PREVAIL (NCT01212991) trial, enzalutamide demonstrated a significant clinical benefit in chemotherapy-naïve patients with mCRPC. At the prespecified interim analysis, enzalutamide in combination with ADT significantly reduced the risk of radiographic progression or death by 68% (HR = 0.32; 95% CI: 0.28–0.37; *p* < 0.0001) and the risk of death by 23% (HR = 0.77; 95% CI: 0.67–0.88; *p* = 0.0002), compared to placebo plus ADT [63]. Median rPFS was 20.0 months with enzalutamide versus 5.4 months with placebo, while median OS was 35.3 months and 31.3 months, respectively [64]. The most frequently reported adverse events associated with enzalutamide were fatigue, back pain, constipation, and arthralgia [65].

In the phase II, randomized, double-blind TERRAIN trial, enzalutamide demonstrated superior efficacy over bicalutamide (50 mg/day) in patients with metastatic castration-resistant PCa. Enzalutamide significantly prolonged PFS compared to bicalutamide (15.7 vs. 5.8 months; HR = 0.44; *p* < 0.0001) [66]. The TITAN (NCT02489318) trial was a multinational, randomized, phase III, double-blind study designed to evaluate the efficacy of adding apalutamide to ADT in patients with mCSPC. A total of 1,052 patients were randomized 1:1 to receive either apalutamide (240 mg once daily) or placebo, both in combination with ADT [67]. Following unblinding in January 2019, patients in the placebo arm were allowed to cross over to apalutamide treatment. With a median follow-up of 44.0 months, a total of 405 OS events were reported, and 208 patients (39.5%) initially assigned to placebo subsequently crossed over to receive apalutamide [68]. (Apalutamide plus ADT significantly reduced the risk of death by 35% compared to placebo plus ADT (median OS not reached vs. 52.2 months; HR = 0.65; 95% CI: 0.53–0.79; *p* < 0.0001), and by 48% after adjustment for crossover (HR = 0.52; 95% CI: 0.42–0.64; *p* < 0.0001) [69]. Apalutamide plus ADT significantly delayed radiographic progression and castration resistance (rPFS; HR = 0.48; 95% CI: 0.39–0.60; *p* < 0.001) [70]. Treatment benefit was consistently observed across all prespecified subgroups, including patients with low-volume disease (37%), those with de novo metastatic presentation (16%), and those with prior localized treatment (1%). Treatment was well tolerated, with a manageable safety profile and preservation of health-related quality of life [71,72].

The CHART trial was a randomized, open-label, phase III study conducted predominantly in China (approximately 90% of enrollment), evaluating the efficacy of rezvilutamide in combination with ADT versus ADT plus bicalutamide in patients with high-volume de novo mPCa [73]. Between 2018 and 2020, 654 patients were randomized to receive either rezvilutamide or bicalutamide, both in combination with ADT. In the pre-specified interim analysis for rPFS, conducted at a median follow-up of 21.2 months (IQR 16.6–25.8), rezvilutamide demonstrated a statistically significant improvement in rPFS compared to bicalutamide (HR = 0.44; 95% CI: 0.33–0.58; *p* < 0.0001) [74]. Similarly, in the interim analysis of OS, at a median follow-up of 29.3 months (IQR 21.0–33.3), rezvilutamide significantly prolonged OS versus bicalutamide (HR = 0.58; 95% CI: 0.44–0.77; *p* = 0.0001) [74].

The phase III ARASENS trial evaluated the addition of darolutamide to standard therapy with ADT and docetaxel in mPCa patients, demonstrating a statistically significant improvement in OS (HR 0.68), with a similar adverse event profile across treatment arms [75]. The survival benefit was consistent across subgroups, including those with high- and low-volume or high- and low-risk disease [75]. The phase III ARANOTE trial showed that darolutamide plus ADT significantly improved radiographic progression-free survival in patients with HSPCa, reducing the risk of progression or death by 46% compared to placebo (HR = 0.54; *p* < 0.0001) [76]. Consistent benefits were observed across disease volume subgroups. While OS favored darolutamide (HR = 0.81), significant improvements were seen in secondary endpoints, including time to castration resistance (HR = 0.40) and time to pain progression (HR = 0.72) [77].

### 2.3. Use of Chemotherapy in Metastatic Prostate Cancer

Patients with mHSPC exhibit heterogeneous prognoses when treated with ADT alone, and their responses to the addition of docetaxel vary accordingly. Individuals presenting with de novo or metachronous high-volume disease derive a significant OS benefit from the combination of docetaxel and ADT [78]. In contrast, patients with synchronous low-volume disease experience only a modest survival advantage, while those with metachronous low-volume disease show no demonstrated survival benefit from docetaxel [78]. The incorporation of molecular or genomic biomarkers may enhance treatment stratification beyond traditional volume-based criteria.

In the setting of mCRPC, both docetaxel and cabazitaxel have been shown to confer OS benefits [79]. The choice of chemotherapeutic agent in mCRPC should be guided by prior treatments administered during the mHSPC phase. Docetaxel is the standard first-line chemotherapy for castration-resistant patients who have not previously received it in the hormone-sensitive stage [79]. In those previously treated with docetaxel, cabazitaxel is the preferred subsequent option, whereas cabazitaxel can also be considered as first-line chemotherapy in select cases where re-treatment with docetaxel is not indicated (see Table 2).

#### 2.3.1. Docetaxel

Docetaxel is a chemotherapeutic agent belonging to the taxane class of anti-mitotic drugs. This class of drugs selectively binds to β-tubulin, inhibiting microtubule dynamics, which disrupts cell division and subsequently induces apoptosis. As an FDA-approved treatment, docetaxel has become the standard of care for several cancer types, including advanced PCa [80]. In 2004, docetaxel became the first therapeutic agent demonstrated to improve both OS and quality of life in patients with mCRPC, conferring a median survival benefit of approximately three months [81,82]. However, its clinical efficacy remains limited, with objective response rates (ORR) reported at only 12%, compared to 7% in patients treated with mitoxantrone (MIT) in the pivotal trial by Tannock et al. [83].

The phase III TROPIC trial evaluated the efficacy of cabazitaxel (25 mg/m^2^) versus mitoxantrone in patients with mCRPC) who had experienced disease progression following prior docetaxel-based chemotherapy [84]. Treatment with cabazitaxel resulted in a median OS of 15.1 months, compared to 12.7 months in the mitoxantrone group (hazard ratio [HR], 0.70; 95% confidence interval [CI], 0.59–0.83; *p* < 0.0001) [84].

#### 2.3.2. Androgen Deprivation Therapy + Docetaxel

Interestingly, several studies have also reported that switching corticosteroids from prednisone to dexamethasone upon disease progression may elicit additional PSA responses in selected patients, suggesting a complex interaction between hormonal manipulation and chemotherapy [85].

In the pivotal TAX 327 trial, patients with mCRPC were randomized to receive mitoxantrone, docetaxel administered every three weeks (75 mg/m^2^), or weekly docetaxel (30 mg/m^2^) [81]. The three-weekly docetaxel regimen significantly improved OS compared to mitoxantrone plus prednisone (hazard ratio [HR] 0.76; 95% confidence interval [CI] 0.62–0.94; *p* = 0.009) [81].

Similarly, in the SWOG 99-16 trial, patients treated with docetaxel (60 mg/m^2^) in combination with estramustine achieved a longer median OS than those receiving mitoxantrone and prednisone (17.5 vs. 15.6 months; HR 0.80; 95% CI 0.67–0.97; *p* = 0.02) [82].

Following these results, the role of docetaxel was subsequently explored in mCSPC. The GETUG-AFU 15 trial did not demonstrate a statistically significant OS benefit with upfront docetaxel in mCSPC; this finding may be partially explained by the high rate of docetaxel use at progression in the ADT alone arm (79%), with a median time to salvage chemotherapy of 18.5 months [25].

In contrast, the CHAARTED trial reported a significant improvement in median OS with the addition of docetaxel to ADT (57.6 vs. 44.0 months; HR 0.61; 95% CI 0.47–0.80; *p* < 0.001), particularly among patients with high-volume disease, defined as the presence of visceral metastases or ≥4 bone lesions with at least one beyond the axial skeleton. The STAMPEDE trial further confirmed the OS benefit of combining docetaxel with ADT in patients with mCSPC [20].

Based on these findings, upfront docetaxel in combination with ADT has become a standard of care in patients with mCSPC, particularly in those with a high disease burden [86]. Retrospective data from GETUG-AFU 15 suggest that rechallenge with docetaxel after progression to mCRPC may retain some activity, although limited to a subset of patients [86]. A ≥50% decline in PSA following first- or second-line docetaxel for mCRPC was observed in 45% of patients who had received initial ADT alone, compared to only 14% among those previously treated with upfront ADT plus docetaxel [86].

In light of the heterogeneity of mHSPC, an evidence-based stratified approach to treatment sequencing between docetaxel (DOC) and ARATs should be guided primarily by tumor burden and patient fitness [86]. For high-volume disease—defined by the CHAARTED criteria as the presence of visceral metastases or ≥4 bone lesions with at least one beyond the axial skeleton—ADT combined with docetaxel remains a preferred option in fit patients requiring rapid cytoreduction or symptom relief, as demonstrated in CHAARTED and STAMPEDE. Alternatively, ADT plus an ARAT (abiraterone, enzalutamide, or apalutamide) offers comparable OS benefits with lower acute toxicity, as supported by LATITUDE and STAMPEDE [86]. In de novo high-volume cases, triple therapy (ADT + DOC + abiraterone) may further improve outcomes, according to the PEACE-1 trial. Conversely, for low-volume disease, the combination of ADT and an ARAT is the treatment of choice, given the lack of survival benefit from upfront docetaxel in this subgroup [86]. The sequencing of DOC and ARAT should be individualized: DOC → ARAT may be favored when rapid disease control is required, whereas ARAT → DOC is preferable when the priority is durable disease control with lower early toxicity. Clinical decisions should further incorporate patient comorbidities, performance status, symptom burden, treatment accessibility, and preferences [86].

#### 2.3.3. Cabazitaxel

Following progression on DOC, cabazitaxel (CAB) emerged as a second-line agent [87]. Cabazitaxel is a microtubule inhibitor belonging to the taxanes family. In vitro studies showed that all taxanes hav a certain cross resistance, but cabazitaxel showed the lowest rate of cross-resistance compared to paclitaxel and docetaxel. This important property justifies the use of cabazitaxel for the treatment of mCRPC previously treated with a docetaxel-containing treatment regimen [87].

In 2010, cabazitaxel—a second-generation taxane—received FDA approval for the treatment of mCRPC in patients previously treated with docetaxel [88].

This approval was supported by the phase III TROPIC trial, which randomized 755 men with mCRPC, whose disease had progressed during or after docetaxel-based therapy, to receive either cabazitaxel 25 mg/m^2^ or mitoxantrone 12 mg/m^2^ every three weeks, both in combination with daily prednisone (10 mg) [84]. The primary endpoint was OS; secondary endpoints included PFS, tumor response, and safety [84]. Cabazitaxel significantly prolonged OS compared to mitoxantrone, with a median OS of 15.1 months (95% CI: 14.1–16.3) versus 12.7 months (95% CI: 11.6–13.7), corresponding to a hazard ratio (HR) of 0.70 (95% CI: 0.59–0.83; *p* < 0.0001) [84]. Median PFS was also improved with cabazitaxel (2.8 months; 95% CI: 2.4–3.0) compared to mitoxantrone (1.4 months; 95% CI: 1.4–1.7), with an HR of 0.74 (95% CI: 0.64–0.86; *p* < 0.0001) [84]. Furthermore, cabazitaxel was associated with a higher objective tumor response rate (14.4% vs. 4.4%, *p* = 0.0005) and a longer time to tumor progression (8.8 vs. 5.4 months, *p* < 0.0001) [84].

The most common adverse events with cabazitaxel were hematologic toxicities, including neutropenia, leukopenia, and anemia. Febrile neutropenia was reported in 8% of patients, and neutropenia-related mortality occurred in 2% (*n* = 7) of those treated with cabazitaxel [78].

#### 2.3.4. Platinum-Based Chemotherapy

While docetaxel remains the standard first-line chemotherapeutic agent for mCRPC, cabazitaxel represents a validated second-line option and may also be considered in selected patients ineligible for docetaxel therapy.

Platinum-based compounds exert their antineoplastic activity primarily through the formation of covalent adducts with cellular DNA, leading to DNA damage predominantly during the G2 phase of the cell cycle and ultimately inducing cell death [89].

However, current evidence suggests that only a limited proportion—estimated between 1% and 10%—of intracellular cisplatin is able to reach the nucleus and interact directly with DNA, thereby triggering cell cycle arrest and apoptosis, particularly in rapidly dividing tumor cells [90].

Cisplatin, the first platinum-containing chemotherapeutic agent, was discovered in the late 1960s and subsequently approved for clinical use in oncology in 1978 [91]. Its efficacy has been well documented across a broad spectrum of malignancies, including ovarian, breast, and gastrointestinal cancers. Despite its potent antitumor activity, long-term administration of cisplatin is frequently associated with significant off-target effects and systemic toxicities, most notably myelosuppression, neurotoxicity, nephrotoxicity, and ototoxicity, which can result in substantial damage to healthy tissues [92].

Platinum-based chemotherapy has shown antitumor activity in mCRPC, although it has not consistently translated into a significant OS benefit in large-scale clinical trials [93]. Emerging evidence suggests that the therapeutic efficacy of platinum compounds, such as cisplatin and carboplatin, may be modulated by the integrity of DNA damage repair (DDR) pathways [94].

Notably, mCRPC tumors harboring BRCA2 mutations appear to exhibit enhanced sensitivity to platinum-based regimens [95,96].

Additionally, the aggressive variant prostate cancer (AVPC) subtype—characterized by clinical aggressiveness and molecular overlap with platinum-responsive small-cell prostate carcinoma—is often defined by concomitant alterations in at least two of the following tumor suppressor genes: TP53, RB1, and PTEN [97]. In a phase 1–2 clinical trial, Corn et al. investigated the combination of cabazitaxel and carboplatin in patients with mCRPC [98]. The addition of carboplatin significantly improved median PFS, extending it from 4.5 to 7.3 months (hazard ratio [HR] 0.69; 95% confidence interval [CI], 0.50–0.95; *p* = 0.018) [98]. Subgroup analyses revealed that this benefit was predominantly confined to patients exhibiting AVPC molecular features, while those lacking such features did not experience a meaningful PFS advantage from the combined regimen [99].

Taxane–platinum combinations have demonstrated encouraging antitumor activity in mCRPC in single-arm clinical trials; however, these results have not been consistently replicated in randomized studies [92].

For example, the RECARDO trial—a randomized phase II study comparing docetaxel monotherapy with docetaxel plus carboplatin in patients with CRPC who had progressed following an initial response to docetaxel—failed to show significant differences in PFS or OS between treatment arms [83].

More recently, a phase I/II randomized trial assessed the efficacy of cabazitaxel in combination with carboplatin in men with progressive mCRPC [98]. The addition of carboplatin to cabazitaxel resulted in improved clinical outcomes compared to cabazitaxel alone, with a median PFS of 7.3 months versus 4.5 months, respectively, at a median follow-up of 31.0 months [92]. Although the combination regimen was associated with a higher incidence of adverse events—including grade 3–5 fatigue, anemia, neutropenia, and thrombocytopenia—it was generally well-tolerated, and no treatment-related deaths were observed [92]. These results support a potential role for taxane–platinum doublets in the management of advanced PCa, warranting further investigation in larger, randomized phase III trials [92].

As a result, investigational efforts have increasingly focused on platinum-based agents—such as cisplatin, carboplatin, and satraplatin—as potential treatment options. Although early-phase trials have shown encouraging signals, their role in the therapeutic landscape of mCRPC remains to be clearly defined [83].

### 2.4. Use of Radiopharmaceuticals in Metastatic Prostate Cancer: II and III Lines of Therapy

Radiopharmaceuticals represent effective treatment options in the advanced stages of mCRPC. Radium-223 was approval by the U.S. Food and Drug Administration (FDA) in 2013 for the treatment of mCRPC to patients who have received at least two lines of systemic treatment for CRPC (abiraterone/enzalutamide and docetaxel) or who are ineligible for these treatments [100]. Lu-PSMA-617 was approved by the U.S. Food and Drug Administration (FDA) in March 2022 for the treatment of mCRPC in patients who have previously received chemotherapy and are no longer responsive to androgen deprivation therapy [101]. These agents offer survival and symptomatic benefits in carefully selected patients.

#### 2.4.1. Radium-223

This radiopharmaceutical selectively targets areas of increased bone remodeling, making it particularly effective in the management of bone metastases associated with PCa [102]. Owing to its alpha-emitting decay characteristics, radium-223 delivers highly localized cytotoxic radiation, resulting in reduced off-target effects on surrounding healthy tissues [103]. This form of radiation induces double-strand breaks in DNA, which is recognized as the primary cytotoxic mechanism underlying the therapeutic action of radium-223 [104].

In the phase III double-blind clinical trial, ALSYMPCA, 921 patients with mCRPC in 2013, were randomised to six injections of 50 kBq/kg radium-223 or placebo as a later-line treatment following the failure or completion of standard therapies [105]. Radium-223 significantly improved OS by 3.6 months in mCRPC patients (hazard ratio [HR]: 0.70, *p* < 0.001), also delaying skeletal events and enhancing pain control and quality of life [106]. Its toxicity was mild, with only slight increases in hematologic effects and diarrhea compared to placebo [106].

The Phase 3 ERA 223 trial enrolled chemotherapy-naïve patients with CRPC and asymptomatic or minimally symptomatic bone metastases, who were randomized to receive abiraterone either in combination with radium-223 or with placebo [107]. The trial was terminated early due to a higher incidence of fractures and mortality observed in the radium-223 arm compared to the control group. Furthermore, the study failed to achieve its primary endpoint—symptomatic skeletal event-free survival—in the intention-to-treat analysis. As a result, the concurrent use of radium-223 and abiraterone is contraindicated [104].

Ricci et al. described a case in which a patient with mCRPC achieved a complete response following treatment with radium-223. Upon disease progression 15 months later, the patient exhibited features of mCSPC and responded to hormone therapy [108]. This case highlights the potential for radium-223 to induce a shift in castration status, suggesting that androgen sensitivity may exist along a dynamic spectrum and could be modulated by radiopharmaceutical therapy, potentially improving prognosis [108].

In the first study by Osvaldo et al., seven mCSPC patients received six cycles of radium-223 in combination with ADT under a compassionate use program [109]. The treatment was generally well tolerated, with expected hematologic effects, such as moderate declines in neutrophil counts and hemoglobin levels. Pain reduction was observed, suggesting a potential symptomatic benefit from radium-223 beyond ADT alone [109]. Notably, imaging with 18F-NaF PET/CT revealed marked changes in standardized uptake values (SUV) in four patients after only three treatment cycles, implying a possible predictive role of this imaging modality in assessing early response to radium-223 [110]. However, due to the concurrent use of ADT, these results remain hypothesis-generating.

In a separate small case series by Wenter et al., 10 mCSPC patients with extensive bone metastatic involvement were treated with radium-223 alongside ADT (without docetaxel or abiraterone) [111]. Seven patients completed all six cycles. Of these, three achieved a complete response and four had stable disease, as determined by bone scintigraphy. Despite the high metastatic burden, the combination therapy showed clinical benefit in several patients. These findings suggest that radium-223, when used with ADT, may offer a therapeutic response comparable to that of more intensive agents like docetaxel or abiraterone, but with a more favorable side-effect profile and potentially better quality of life. Nonetheless, the confounding effect of ADT responsiveness must be considered [112].

The ADDRAD trial is an ongoing phase II study investigating the safety and efficacy of combining radium-223 with definitive EBRT to the prostate in patients with mCSPC, who are managed non-surgically [113]. In this protocol, patients receive concurrent whole-pelvis EBRT (74 Gy to the prostate and 60 Gy to pelvic lymph nodes) along with radium-223. A total of 30 patients are planned for enrollment, with 20 accrued to date [20]. Early results from the first 10 patients, presented in 2018, reported a low incidence (4.2%) of grade 3 adverse events, and seven patients showed partial response on whole-body MRI [113]. The trial also allows prior administration of docetaxel, enabling future comparison of outcomes such as biochemical control and progression-free survival with historical data from trials like CHAARTED, which assessed docetaxel alone [26].

The concept of integrating radium-223 into oligometastatic mCSPC treatment was first proposed by researchers at City of Hope in 2015, following publication of the ALSYMPCA trial [114]. Building on this, their current phase II trial (NCT#03361735) combines SBRT, radium-223, and short-course ADT. The primary endpoints include time to treatment failure (based on PSA criteria) and objective response rate. Preclinical evidence suggests ADT may radiosensitize PCa cells, justifying its inclusion in the regimen. To date, four patients have completed six cycles of radium-223 (as in ALSYMPCA), and a fifth completed the same regimen off protocol [114]. Feasibility data from these cases will be published in the future. A second trial, RROPE (NCT#03304418), is underway at the University of Utah, exploring radium-223 with SBRT in hormone-naïve men with oligometastatic bone involvement, without systemic therapy. Key endpoints include time to ADT initiation, quality of life, and OS [115]. These outcomes will be compared to historical SBRT-only studies like STOMP [116]. A third study, the RAVENS trial (NCT#04037358) at Johns Hopkins, is a randomized phase II trial assessing radium-223 plus SBRT vs. SBRT alone in patients with oligometastatic PCa [117]. This trial does not permit concurrent ADT, focusing instead on progression-free survival as the main endpoint [118]. Collectively, these three ongoing studies aim to evaluate the safety, feasibility, and potential synergistic effect of combining radium-223 (a systemic radiopharmaceutical) with SBRT (a focal ablative therapy) in the treatment of oligometastatic PCa [117]. The results may help define whether dual radiation strategies can improve disease control compared to SBRT alone [119].

Final data on efficacy and safety from the EORTC 1333/PEACE III trial are still pending and are expected to clarify how the combination of radium-223 (Ra-223) and enzalutamide may be optimally used for the treatment of mCRPC [120]. Preclinical evidence suggests that combining Ra-223 with enzalutamide may offer enhanced antitumor activity compared to either agent alone, and potentially without compromising bone health [121]. Preliminary safety data from the trial indicate that when bone health agents (BHAs) are co-administered, the risk of fractures with the Ra-223 and enzalutamide combination is comparable to enzalutamide monotherapy [104]. Moreover, the combination has demonstrated an acceptable safety profile in phase II trials [120].

#### 2.4.2. Lu-PSMA-617

Lutetium-177–labeled PSMA-617 (Lu-PSMA-617) is a targeted radioligand therapeutic agent that combines a prostate-specific membrane antigen (PSMA)-binding peptidomimetic with the β-emitting radionuclide Lutetium-177 [122]. This compound enables the selective delivery of ionizing radiation to PSMA-expressing PCa cells, inducing DNA damage and cell death while minimizing exposure to surrounding healthy tissues [122]. Marketing authorization in the United Kingdom followed in August 2022. PSMA-617 demonstrates high specificity for PSMA, a transmembrane glycoprotein markedly overexpressed in PCa cells but with limited expression in most normal tissues [101]. This differential expression facilitates high tumor uptake with minimal off-target accumulation, particularly in non-prostatic organs, thereby reducing systemic toxicity and enhancing the safety profile of the therapy [123].

The development of PSMA-targeted agents has been an area of active research, particularly for diagnostic imaging applications using positron emission tomography (PET). Urea-based low-molecular-weight PSMA inhibitors were first introduced in preclinical models in 2005 and have since shown high tumor affinity [124,125]. However, early compounds often exhibited significant renal uptake, which presented challenges related to unfavorable pharmacokinetics and potential nephrotoxicity [126].

Initial clinical experience with PSMA-617 began in 2014, demonstrating its potential for both diagnostic imaging and radioligand therapy in advanced PCa—a strategy now referred to as theranostics [127,128]. This approach integrates molecular diagnostics with targeted therapy, allowing for personalized treatment based on tumor-specific biomarker expression [129]. Theranostic strategies not only enable patient stratification but also facilitate real-time monitoring of treatment response, ultimately aiming to enhance therapeutic efficacy and safety [128]. In a radiopharmaceutical theranostic approach, the patient first undergoes molecular imaging using a targeted PET tracer. This diagnostic step enables visualization of the biodistribution and specific accumulation of the radiotracer at the target site, such as a receptor overexpressed by a particular tumor type [129]. If the primary tumor and potential metastases exhibit adequate uptake of the labeled biomarker, the patient may then receive endoradiotherapy with a therapeutic radiopharmaceutical that shares an identical or closely related molecular structure to the diagnostic agent. Therapy response can subsequently be monitored through follow-up imaging using the corresponding PET tracer [130].

In this phase 2, open-label, randomized multicenter trial (TheraP trial 2021), [^177^Lu]Lu-PSMA-617 was evaluated against cabazitaxel in men with mCRPC who had previously received AR–targeted therapy and were eligible for cabazitaxel as the next standard treatment [131]. Of 291 men screened, 200 met PET eligibility criteria—defined as PSMA-positive disease on [^68^Ga]Ga-PSMA-11 PET and absence of discordant FDG-positive/PSMA-negative metastatic sites on [^18^F]FDG PET [132]. Ninety-nine patients were randomized to receive up to six cycles of [^177^Lu]Lu-PSMA-617 (6.0–8.5 GBq every 6 week), and 101 to receive up to ten cycles of cabazitaxel (20 mg/m^2^ every 3 weeks). PSA response, defined as a ≥50% reduction from baseline, was significantly higher in the [^177^Lu]Lu-PSMA-617 group: 66% (65 of 98 patients treated) vs. 37% (37 of 101) in the cabazitaxel group by intention-to-treat analysis (absolute difference 29%, 95% CI 16–42; *p* < 0.0001), and 66% vs. 44% among those who received treatment (difference 23%, 95% CI 9–37; *p* = 0.0016) [133]. In terms of safety, grade 3–4 adverse events occurred in 33% of patients receiving [^177^Lu]Lu-PSMA-617, compared to 53% in the cabazitaxel group, indicating a more favorable toxicity profile. Importantly, no deaths were attributed to [^177^Lu]Lu-PSMA-617 [134]. These findings demonstrate that [^177^Lu]Lu-PSMA-617 not only yields a significantly higher PSA response compared to cabazitaxel, but also results in fewer severe treatment-related adverse events, highlighting its potential as a more effective and better-tolerated therapeutic option for patients with advanced PSMA-positive mCRPC [131]. The VISION trial (NCT03511664) was an international, open-label, phase 3 study designed to assess the efficacy and safety of [^177^Lu]Lu-PSMA-617 in patients with mCRPC who had previously received at least one AR pathway inhibitor and one or two taxane-based chemotherapy regimens [135]. Eligibility was confirmed via gallium-68–labeled PSMA-11 PET/CT, with inclusion restricted to individuals demonstrating PSMA-positive disease. Participants were randomized in a 2:1 ratio to receive [^177^Lu]Lu-PSMA-617 (administered at 7.4 GBq every 6 weeks for 4 to 6 cycles) in addition to protocol-permitted standard of care (SOC), or SOC alone [136]. Between June 2018 and mid-October 2019, 831 of the 1,179 screened patients were randomized [136]. The median duration of follow-up was 20.9 months. Treatment with [^177^Lu]Lu-PSMA-617 in combination with standard therapy resulted in a statistically significant improvement in both imaging-based progression-free survival (median: 8.7 vs. 3.4 months; hazard ratio [HR] for progression or death: 0.40; 99.2% confidence interval [CI]: 0.29–0.57; *p* < 0.001) and OS (median: 15.3 vs. 11.3 months; HR for death: 0.62; 95% CI: 0.52–0.74; *p* < 0.001), compared to SOC alone. All key secondary endpoints also significantly favored the [^177^Lu]Lu-PSMA-617 arm. While the incidence of grade ≥3 adverse events was higher in the [^177^Lu]Lu-PSMA-617 group (52.7% vs. 38.0%), health-related quality of life was not adversely impacted, indicating that the clinical benefits of the radioligand therapy were achieved without a detrimental effect on patient-reported outcomes [137]. [^177^Lu]Lu-PSMA-617 demonstrated an overall favorable safety profile, with renal radiotoxicity remaining minimal and not constituting a significant safety concern [138].

In the phase 2 TheraP trial, patients with advanced mCRPC, who had previously undergone first-line treatment with chemotherapy and ARPIs, were randomly assigned in a 1:1 ratio to two treatment arms [139]. One cohort received up to six cycles of radioligand therapy (RLT) with Lu-PSMA-617, while the comparator arm was administered up to ten cycles of cabazitaxel-based chemotherapy [140]. The primary endpoint of the study was the PSA response rate. Secondary endpoints included PFS, OS, and treatment-related toxicity [141]. A total of 101 participants received cabazitaxel, while 99 were treated with Lu-PSMA-617. A PSA reduction of ≥50% was observed in 66% of patients in the Lu-PSMA-617 arm, compared to 37% in the cabazitaxel arm (*p* < 0.0001) [141]. However, a subsequent publication from the same research group indicated no statistically significant difference in OS between the two cohorts. Notably, the Lu-PSMA-617 treatment group exhibited a substantially lower incidence of adverse events, and patients receiving RLT reported superior improvements in health-related quality of life compared to those treated with cabazitaxel [141].

The PSMAfore trial, a randomized phase III study evaluating the efficacy of Lu-PSMA-617 compared to a switch in ARPI therapy in chemotherapy-naïve patients with mCRPC, was recently presented at the European Society for Medical Oncology (ESMO) [142]. The investigators reported a significantly prolonged rPFS in the Lu-PSMA-617 arm, with a median duration of 12.2 months, compared to 5.9 months in the ARPI switch arm [142]. The high crossover rate (84%) will complicate the analysis of OS at later follow-up stages [143].

### 2.5. Use of Target Therapy in Metastatic Prostate Cancer

Over the past decade, the therapeutic landscape of PCa has undergone a significant transformation with the introduction of new cytotoxic agents and ARTA. Randomized controlled trials (RCTs) have shown substantial improvements in oncological outcomes when ARTA are combined with ADT. Initially approved only for mCRPC, these treatment regimens are now also indicated for use in the mHSPC [59,144].

The main goal is to delay or prevent the progression to castration-resistant disease, the terminal stage marked by the onset of symptoms, treatment-related adverse events, significant deterioration in quality of life, and a heightened risk of mortality [145].

PCa involves both germline and somatic genetic alterations; germline mutations, present in all body cells, are relevant for genetic counseling, while somatic mutations arise specifically in tumor cells and may result from intrinsic genomic instability and clonal selection following previous treatments [146]. Germline mutations in genes involved in homologous recombination (HR) DNA repair pathways have been identified in approximately 10–15% of patients with metastatic PCa, while somatic mutations in these same pathways are observed in 20–25% of cases, with BRCA2 and ATM being the most commonly affected genes [147]. Additionally, compared to locoregional tumors, mCRPC exhibits a higher prevalence of alterations in AR, TP53, RB1, and PTEN genes [148].

#### 2.5.1. PARP-Inhibitors

During its biological lifespan, the genome is constantly subjected to various types of damage caused by both external factors—such as ultraviolet radiation from sunlight, genotoxic chemicals, and ionizing radiation—and internal sources, including reactive oxygen species, abasic sites, and base deamination events that lead to miscoding. To manage these diverse DNA lesions, eukaryotic cells rely on a complex network of precisely coordinated defense mechanisms. These include damage recognition and signaling pathways, chromatin remodeling, DNA repair systems, and cell-cycle checkpoints [149].

Targeting DNA damage repair (DDR) pathways represents a rapidly evolving approach in cancer therapy. DNA damage induces genomic instability, making cells increasingly dependent on DDR mechanisms for survival. In response to such damage, DDR signaling activates the transcription of specific repair proteins, resulting in their overexpression and the subsequent initiation of DNA repair processes [150].

**Figure 1:**
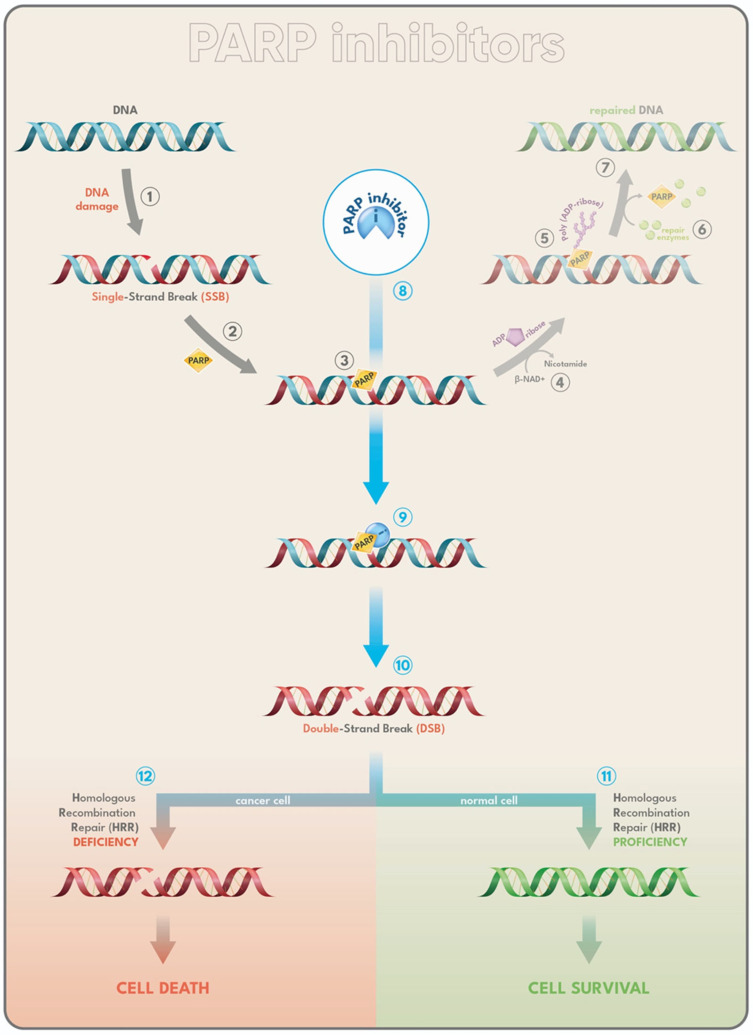
Mechanism of Action of PARP Inhibitors. **DNA damage and PARPs**. The most frequent form of DNA damage is the single-strand break (SSB) (1), which is primarily repaired through the PARP-dependent base excision repair (BER) pathway (2). Members of the PARP (Poly ADP-Ribose Polymerase) enzyme family catalyze the transfer of ADP-ribose units to target proteins. Some PARP isoforms act as sensors that detect SSBs and initiate an immediate repair response. Upon recognizing a SSB, a PARP enzyme binds to the damaged DNA site (3) and undergoes a conformational shift that enables β-NAD⁺, its required cofactor, to access the active site (4). PARP then cleaves β-NAD⁺ and transfers ADP-ribose units to target proteins, generating a poly(ADP-ribose) (PAR) chain (5). This modification promotes the recruitment of DNA repair factors via their PAR-binding domains (6), allowing the repair machinery to efficiently resolve the lesion (7). The repair process concludes with PAR degradation by Poly(ADP-ribose) Glycohydrolase (PARG) and release of PARP and associated enzymes. **PARP inhibitors**. PARP inhibitors (PARPi) (8) block PARP catalytic activity, preventing PARylation, and trap PARP molecules on damaged DNA (9). When SSBs cannot be repaired, they are converted into double-strand breaks (DSBs) during DNA replication, leading to additional DNA damage under PARP inhibition (10). DSBs can be repaired through homologous recombination (HR), so HR-proficient cells are able to survive by correcting these lesions (11). In contrast, HR-deficient cells—such as those harboring BRCA1 or BRCA2 mutations—cannot repair DSBs and undergo cell death (12). Because many tumors carry defects in DNA repair pathways, PARP inhibitors exploit the principle of *synthetic lethality*, where two individually non-lethal defects become lethal when combined.

The accumulation or inefficient repair of DNA damage is a key contributor to the emergence of mutations, which are central to the initiation and progression of most cancers. To preserve genomic stability, healthy cells activate a coordinated network of signaling pathways as DDR. These pathways detect DNA lesions, pause the cell cycle, and trigger appropriate repair mechanisms. Among the primary mediators of the DDR are the enzymes Poly(ADP-ribose) Polymerase 1 and 2 (PARP1 and PARP2), which function as DNA damage sensors and signal transducers [151]. When PARP1 recognizes DNA damage—particularly single-strand breaks (SSBs)—it undergoes conformational changes that activate its catalytic domain [149]. This activation initiates the synthesis of negatively charged, branched poly(ADP-ribose) (PAR) chains on various protein targets, a process known as PARylation [151]. This modification promotes the recruitment of DNA repair proteins and facilitates chromatin remodeling around the lesion, thereby enabling efficient repair as schematically illustrated in Figure 1 [151]. As part of the regulatory cycle, PARP1 also modifies itself through autoPARylation; the accumulation of negative charges on the enzyme is thought to weaken its DNA binding affinity, promoting its release from the repaired site [151].

PARP inhibitors (olaparib, rucaparib, niraparib, and talazoparib) are a prime example of precision medicine, as they selectively target cancer cells with DNA repair deficiencies, such as BRCA1/2 mutations, leading to increased genomic instability and cell death [152].

Inhibiting PARP-dependent repair of DNA damage induced by chemotherapy or radiotherapy has the potential to enhance the cytotoxic effectiveness of these treatments. Approximately three decades ago, small-molecule nicotinamide analogs were found to suppress PARylation and increase the cell-killing effects of dimethyl sulfate, a known DNA-damaging agent [153].

Individuals with harmful heterozygous germline mutations in the BRCA1 or BRCA2 genes face a markedly increased risk of developing breast, ovarian, and prostate cancers [154].

Since the wild-type BRCA allele is typically lost during tumor development, BRCA1 and BRCA2 are classified as classic tumor suppressor genes. The proteins encoded by these genes play an essential role in repairing DNA double-strand breaks through homologous recombination repair (HRR), a high-fidelity repair pathway that utilizes a homologous DNA template to accurately restore the original DNA sequence at the site of damage [155].

In the absence of functional HRR—whether due to mutations in BRCA1, BRCA2, or other components of the pathway—cells increasingly rely on error-prone DNA repair mechanisms such as Non-Homologous End Joining (NHEJ). These alternative pathways repair double-strand breaks by directly ligating DNA ends without the use of a homologous template, or by joining nearby DNA regions with minimal sequence homology, often resulting in the deletion of the intervening genetic material. This shift toward non-conservative repair frequently leads to genomic alterations, including the loss of DNA sequences [156].

Certain mutations resulting from these error-prone repair processes may contribute to cancer onset or progression, which helps explain, at least in part, the elevated cancer risk associated with BRCA1 and BRCA2 mutations. Moreover, the involvement of BRCA1 and BRCA2 in other cellular functions—such as chromatin remodeling and regulation of gene transcription—may further influence tumor development [157].

The finding that BRCA-mutant tumor cells exhibited up to 1000-fold greater sensitivity to PARP inhibitors compared to BRCA-wild type cells (depending on the specific inhibitor and experimental conditions) served as a strong rationale for evaluating PARPi as standalone therapies in clinical trials [158].

The pivotal study that led to the approval of a PARP inhibitor in pCar was the PROfound trial, which investigated the efficacy of olaparib in men with mCRPC whose disease had progressed during or after treatment with ARATs [159]. The study population was stratified into two cohorts: Cohort A included 245 patients with alterations in BRCA1, BRCA2, or ATM genes, while Cohort B comprised 142 patients with mutations in other predefined genes [159,160]. Participants in both cohorts were randomly assigned to receive either olaparib or the physician’s choice of enzalutamide or abiraterone. In Cohort A, the primary endpoint—imaging-based PFS—was significantly improved in the olaparib group compared to the control group, with a median PFS of 7.4 months versus 3.6 months (hazard ratio for progression or death, 0.34; 95% CI, 0.25–0.47; *p* < 0.001) [159]. Additionally, olaparib demonstrated benefit in imaging-based PFS in the overall study population (Cohorts A and B). Median OS in Cohort A was 18.5 months for patients treated with olaparib compared to 15.1 months in the control group, while in Cohort B, median OS was 14.1 months versus 11.5 months, respectively [159].

Given the significantly greater clinical benefit observed in Cohort A of the PROfound trial, current European guidelines—such as those outlined in the ESMO Clinical Practice Guidelines for pCa recommend the use of olaparib after treatment with novel hormonal agents exclusively in patients with mCRPC who harbor BRCA1 or BRCA2 mutations [100]. This recommendation is supported by robust evidence indicating that BRCA1/2-altered tumors are particularly sensitive to PARP inhibition, whereas the benefit in patients with other DNA repair gene alterations appears to be more limited [100].

In contrast, in the United States, the FDA has approved olaparib for a broader population—specifically for patients with pathogenic germline or somatic mutations in any of several HRR genes, including BRCA1, BRCA2, ATM, BARD1, BRIP1, CDK12, CHEK1, CHEK2, FANCL, PALB2, RAD51B, RAD51C, RAD51D, and RAD54L—regardless of the individual gene’s sensitivity to PARP inhibition [161]. This broader indication reflects the current NCCN Guidelines^®^, which acknowledge the clinical relevance of a wider spectrum of HRR gene alterations, even though the magnitude of benefit may vary depending on the specific mutation involved [161]. However, a major practical limitation in implementing PARP inhibitor therapy is the relatively low detection rate of HRR mutations in clinical practice. Real-world data indicate that only about 20–25% of expected alterations are effectively identified, due to tumor heterogeneity, limited or poor-quality biopsy tissue, and variability across sequencing assays [162]. This means that a proportion of patients who might benefit from PARPi remain undetected. To overcome this, optimized biomarker approaches are needed, including the use of comprehensive next-generation sequencing (NGS) on both tumor tissue and circulating tumor DNA (ctDNA), repeat testing in selected cases, and integration with germline genetic testing [162]. Until more reliable and standardized assays are available, clinicians should be aware of these limitations when recommending PARPi, as they directly affect patient selection and access to treatment [162].

##### Abiraterone-Prednisone Plus Olaparib

The phase III PROpel trial evaluated the efficacy of combining olaparib with abiraterone and prednisone/prednisolone as a first-line treatment for mCRPC, independently of homologous recombination repair (HRR) mutation status [163]. Patients were randomized to receive either the combination therapy or placebo plus abiraterone and prednisone/prednisolone [163]. The addition of olaparib led to a statistically significant improvement in imaging-based progression-free survival (ibPFS), with a median duration of 24.8 months compared to 16.6 months in the control group (hazard ratio 0.66; 95% CI, 0.54–0.81; *p* < 0.001) [163].

Based on these results, the European Union has granted approval for the use of olaparib in combination with abiraterone as a first-line treatment option for mCRPC [163]. In the PROpel study, the combination of olaparib with abiraterone was associated with a higher incidence of treatment-emergent adverse events compared with placebo plus abiraterone. Grade ≥ 3 adverse events occurred in approximately 54–58% of patients receiving olaparib plus abiraterone, versus 42–46% in the control group [164]. Patients were stratified into asymptomatic/mildly symptomatic and symptomatic subgroups, and the toxicity pattern was consistent across both. As shown in Table 3 the most frequent adverse event was anemia, observed in 52.5% of asymptomatic/mildly symptomatic patients (grade ≥ 3 in 14.3%) and 45.2% of symptomatic patients (grade ≥ 3 in 20.2%) in the olaparib arm, compared with 17.0% (grade ≥ 3 in 3.1%) and 22.5% (grade ≥ 3 in 5.0%) in the placebo arms [164]. Other commonly reported events in the olaparib group included fatigue/asthenia (34–46%), nausea (29–35%), diarrhea (16–22%), decreased appetite (10–18%), vomiting (14–18%), hypertension (13–14%), neutropenia (8–14%), and musculoskeletal pain, generally at higher rates than in the control group [164].

A recent meta-analysis including 1361 patients showed that the combination of abiraterone with a PARP inhibitor (such as olaparib) was associated with a significantly increased risk of cardiovascular toxicity of any grade (RR 1.57; 95% CI 1.14–2.17), although no significant increase in high-grade events was demonstrated (RR 3.39; *p* = 0.36) [165]. Conversely, the incidence of hypertension was not significantly higher compared to abiraterone monotherapy [165].

##### Abiraterone-Prednisone Plus Niraparib

The phase III MAGNITUDE trial evaluated the efficacy of combining niraparib with abiraterone acetate and prednisone (AAP) as a first-line treatment for mCRPC, stratifying patients based on the presence or absence of homologous recombination repair (HRR) gene alterations [166]. Among 423 HRR-positive (HRR+) patients, those treated with niraparib plus AAP experienced a significant improvement in rPFS, particularly in the BRCA1/2-mutated subgroup, where median rPFS reached 19.5 months compared to 10.9 months in the placebo group (HR 0.55; 95% CI 0.39–0.78; *p* = 0.0007) [166]. The benefit was consistent across other endpoints, including time to symptomatic progression and time to cytotoxic chemotherapy initiation [167]. Although OS data were immature, adjusted analyses accounting for post-progression treatments suggested a favorable OS trend in BRCA1/2 patients (HR 0.54; 95% CI 0.33–0.90; *p* = 0.0181) [167]. In contrast, patients without HRR alterations (HRR−) showed no clinical benefit from the addition of niraparib, confirming the importance of molecular stratification [167]. The safety profile of the combination was manageable, and no significant differences in patient-reported quality of life were observed [167]. These findings underscore the clinical relevance of identifying HRR alterations—particularly BRCA1/2 mutations—to guide targeted treatment strategies in mCRPC [167,168].

##### Rucaparib

The phase II TRITON2 study evaluated the efficacy of the PARP inhibitor rucaparib in patients with mCRPC harboring BRCA1 or BRCA2 alterations [169]. Patients received rucaparib 600 mg twice daily after progressing on one or two lines of androgen receptor–targeted therapy and one taxane-based chemotherapy [102]. The confirmed ORR ranged from 43% to 51%, while 55% of patients achieved a ≥50% decline in PSA levels [169]. Similar efficacy was observed across germline and somatic BRCA alterations, as well as between BRCA1 and BRCA2 subgroups, with higher PSA responses noted in BRCA2-mutated cases; the most common grade ≥ 3 adverse event was anemia (25%) as shown in Table 3 [169]. However, these results derive from a single-arm trial without a comparator arm and should therefore be interpreted with caution. Nevertheless, TRITON2 provided important preliminary evidence of antitumor activity for rucaparib in BRCA-altered mCRPC, supporting its further evaluation in randomized controlled studies [169].

##### Enzalutamide Plus Talazoparib

The phase III TALAPRO-2 trial evaluated the efficacy of combining talazoparib, a PARP inhibitor, with enzalutamide as a first-line treatment for mCRPC, based on preclinical evidence suggesting synergistic activity between AR blockade and PARP inhibition. The study enrolled 805 patients, regardless of HRR gene alteration status, and included a separate HRR-deficient cohort. Patients were randomized 1:1 to receive talazoparib or placebo, both in combination with enzalutamide. In the overall population, talazoparib plus enzalutamide significantly prolonged rPFS compared to enzalutamide alone (median not reached vs. 21.9 months; HR 0.63; 95% CI 0.51–0.78; *p* < 0.0001). In the HRR-deficient subgroup, the benefit was even more pronounced (HR 0.45; 95% CI 0.33–0.61; *p* < 0.0001). Although OS data were still immature, adjusted analyses suggested a favorable trend for talazoparib. As shown in Table 3 the most common adverse events were anemia, neutropenia, and fatigue, with anemia being the leading grade 3–4 event, manageable with dose adjustments. These findings support talazoparib plus enzalutamide as a promising first-line treatment option for patients with mCRPC, regardless of HRR status, pending confirmation from ongoing long-term follow-up.

The most frequent type of DNA damage is the single-strand break (SSB), typically repaired through the base excision repair (BER) pathway, which depends on the activity of poly(ADP-ribose) polymerases (PARPs).

Certain isoforms within the PARP family act as sensors of SSBs, initiating an immediate cellular response. Upon detecting a break, PARP binds to the damaged DNA, undergoes a conformational change, and facilitates the binding of its cofactor β-NAD^+^ to its active site. Through the hydrolysis of β-NAD^+^, PARP catalyzes the transfer of ADP-ribose units to itself and other target proteins—a process known as PARylation—resulting in the formation of poly(ADP-ribose) chains [151].

These PAR chains act as docking sites for DNA repair effectors, which are recruited to the damage site to restore DNA integrity. Once repair is complete, poly(ADP-ribose) glycohydrolase (PARG) degrades the PAR chains, allowing the release of PARP and other repair proteins. PARP inhibitors (PARPi) interfere with this process by blocking the catalytic activity of PARP (thus preventing PARylation) and trapping the enzyme on DNA, thereby stalling the repair of SSBs. If unresolved, SSBs can evolve into more deleterious double-strand breaks (DSBs) during DNA replication. In cells competent for homologous recombination (HR), DSBs can still be accurately repaired. However, HR-deficient cells—such as those with BRCA1 or BRCA2 mutations—lack this backup repair mechanism. As a result, the accumulation of unrepaired DSBs leads to cell death. This therapeutic strategy leverages the concept of synthetic lethality, where two individually non-lethal defects—PARP inhibition and homologous recombination deficiency—combine to cause selective cancer cell death [151].

#### 2.5.2. PI3K/AKT/mTOR Inhibitors

The PI3K/AKT signaling cascade, together with alterations in the tumor suppressor gene PTEN, is a major driver of PCa biology and a key modulator of AR signaling [170,171].

Over time, PCainevitably evolves toward a castration-resistant state, in which ADT and AR antagonists lose their effectiveness. From a mechanistic perspective, tumor cells adapt by acquiring a more aggressive phenotype and reducing their dependence on AR signaling, while instead activating alternative survival pathways [170,171].

A pivotal mechanism in this adaptation is the hyperactivation of the PI3K–AKT–mTOR pathway, a central regulator of cell survival and resistance to apoptosis that can compensate for AR blockade. This pathway exhibits intricate bidirectional crosstalk with both genomic and non-genomic AR signaling, as well as with other molecular networks, creating a reciprocal feedback loop that complicates the selective targeting of single pathways or molecules in advanced PCa. Importantly, activation of the PI3K pathway is closely linked to the onset of castration resistance, disease progression, and poor clinical prognosis. Notably, AR inhibition can increase AKT phosphorylation, whereas PTEN loss can further suppress AR activity, underscoring the tight regulatory interplay between these two signaling axes [170,171].

In more detail Phosphatidylinositol-3-kinase (PI3K) is a membrane-associated protein kinase that functions as a critical intermediary, linking extracellular signals from growth factors and cytokines to downstream intracellular signaling pathways [172]. Phosphoinositide 3-kinases (PI3Ks) form a large group of lipid kinases, classified into three categories—Class I (further subdivided into IA and IB), Class II, and Class III—based on their substrate specificity and structural organization [173].

Of the three PI3K classes (I–III), class IA is the most frequently associated with human cancers, including PCa [174].

The tumor suppressor PTEN (phosphatase and tensin homolog deleted on chromosome 10) serves as a critical negative regulator of the PI3K-AKT-mTOR signaling pathway [175]. It does so by dephosphorylating PIP3 (phosphatidylinositol (3,4,5)-trisphosphate), converting it back to PIP2. When PIP3 levels are elevated, this promotes the activation of several kinases, including PDK1, which in turn phosphorylates AKT at the Thr308 site [175]. Once activated, AKT phosphorylates a range of downstream targets that control key cellular functions, such as FOXO transcription factors, glycogen synthase kinase 3 beta (GSK3β), NF-κB, and the tuberous sclerosis complex protein TSC2 [176]. For example, phosphorylation of TSC2 by AKT leads to its inactivation, thereby allowing RHEB to stimulate mTORC1 activity [177]. This activation suppresses autophagy and promotes cell growth, protein synthesis, and ribosome production through phosphorylation of mTORC1 effectors like ULK1, S6 kinase (S6K), and 4EBP1 [177].

Additionally, phosphorylated S6K can modulate mTORC2 signaling by phosphorylating RICTOR [178]. mTORC2, in turn, influences cellular survival, progression through the cell cycle, and cytoskeletal organization by targeting molecules such as AKT (at Ser473, further enhancing its activity), serum/glucocorticoid-regulated kinase 1 (SGK1), and protein kinase C alpha (PKCα) [178].

**Figure 2:**
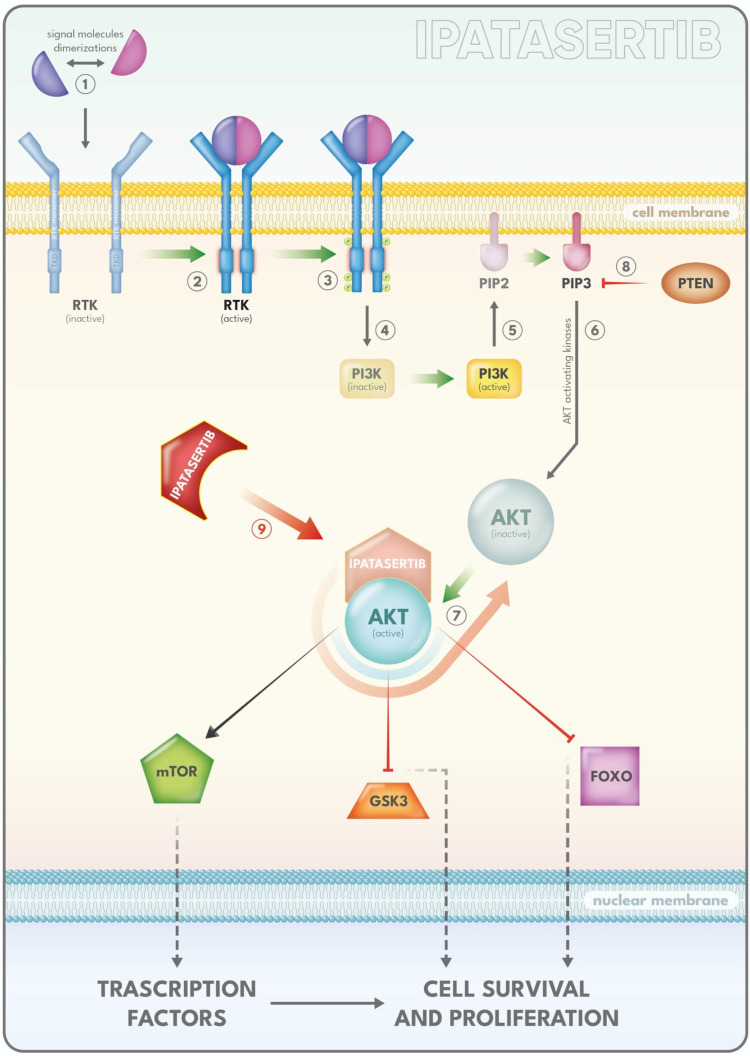
Mechanism of Action of Ipatasertib—PI3K/AKT Signaling Pathway. Many cancers display dysregulation of the PI3K (phosphatidylinositol 3-kinase)/AKT (also known as PKB, protein kinase B) pathway, which governs essential cellular processes such as metabolism, cell-cycle progression, survival, proliferation, motility, and differentiation. Activation of the PI3K/AKT cascade begins with stimulation of a Receptor Tyrosine Kinase (RTK). When ligands bind to the extracellular domain of the RTK (1), two receptor monomers come together to form a dimer (2). Dimerization activates the intracellular tyrosine kinase domains (TKDs), enabling each monomer to phosphorylate multiple tyrosine residues on its partner (3). These phosphotyrosines act as docking sites for downstream signaling proteins, ultimately leading to PI3K phosphorylation and activation (4). Activated PI3K converts PIP2 to PIP3 (5), which facilitates the recruitment and activation (6) of AKT. As the central effector of the pathway, activated AKT regulates numerous cellular functions (7). The pathway is negatively regulated by the tumor suppressor PTEN (8), which dephosphorylates PIP3 and restrains PI3K signaling. Mutations or alterations in PTEN, PI3K, or AKT—commonly found in various cancers—result in persistent AKT hyperactivation and uncontrolled cell growth. Ipatasertib exerts its activity by binding to the ATP-binding pocket of AKT (9), thereby inhibiting its activation and preventing downstream signaling. Through this mechanism, ipatasertib suppresses cellular proliferation and survival.

The dysregulation of the PI3K-AKT-mTOR pathway include gain-of-function mutations or amplifications affecting PI3K, AKT, and/or mTOR themselves [179]. An aberrant activation of the PI3K pathway has been identified in approximately 40% of early-stage PCa and up to 70% of advanced cases [180]. Loss of the tumor suppressor gene PTEN is one of the most frequent genetic alterations observed in PCa, occurring in up to 50% of cases of CRPCa [181]. This pathway is schematically illustreated in Figure 2.

AKT inhibitors are emerging as promising therapeutic agents in the context of precision medicine for the treatment of mCRPC [182].

##### Ipatasertib

Ipatasertib inhibits AKT by binding to its ATP-binding site in a competitive manner, thereby blocking PI3K/AKT signaling as illustrated in Figure 2. This inhibition can suppress tumor cell proliferation and promote apoptosis [182]. The phase 3 IPATential150 trial (NCT03072238) assessed the efficacy of combining ipatasertib with abiraterone in patients with mCRPC exhibiting PTEN loss [183]. Results showed a median rPFS of 18.5 months (95% CI: 16.3–21.0) in the ipatasertib plus apalutamide group, compared to 16.5 months (95% CI: 13.9–17.0) in the placebo plus apalutamide arm [183]. This combination led to a 23% improvement in rPFS (HR: 0.77; 95% CI: 0.61–0.98; *p* = 0.0335) [183]. However, this clinical benefit was restricted to the PTEN-loss subgroup, with no significant improvement in the overall intention-to-treat population and no advantage in OS at the time of the final analysis [183]. Moreover, translational hurdles emerged, including variability across PTEN assays and the occurrence of class-specific toxicities such as diarrhea, hyperglycemia, and rash [184]. These findings highlight that, despite a strong biological rationale, AR–AKT co-inhibition currently provides only incremental benefits, confined to molecularly selected patients [184].

##### Capivasertib

A phase 3 randomized trial, CAPItello-280 (NCT05348577), is currently ongoing to assess the efficacy and safety of combining capivasertib with docetaxel, compared to docetaxel with placebo, in the treatment of patients with mCRPC [185]. In parallel, the CAPItello-281 trial (NCT04493853), also randomized, is evaluating the therapeutic potential of capivasertib—a potent and selective inhibitor of all three AKT isoforms (AKT1, AKT2, and AKT3)—in combination with abiraterone in patients with mCSPC exhibiting PTEN loss [186].

The PI3K/AKT (phosphatidylinositol 3-kinase/protein kinase B) signaling cascade plays a pivotal role in controlling key cellular processes such as metabolism, cell cycle regulation, survival, proliferation, motility, and differentiation. Aberrant activation of this pathway is a defining feature of numerous cancers. The cascade is initiated when extracellular ligands bind to receptor tyrosine kinases (RTKs) (1), triggering dimerization of RTK monomers (2) and activation of their cytoplasmic kinase domains. This leads to autophosphorylation of tyrosine residues (3), which act as docking sites for signaling proteins that, in turn, activate PI3K (4). PI3K converts PIP2 into PIP3 (5), enabling the recruitment and phosphorylation of AKT by upstream kinases (6). Once activated, AKT serves as a central hub that regulates multiple essential cellular activities (7). Under physiological conditions, the tumor suppressor PTEN antagonizes this pathway by dephosphorylating PIP3, thereby preventing AKT activation (8). However, genetic alterations in PTEN, PI3K, or AKT can result in persistent pathway activation, driving uncontrolled cell proliferation. Ipatasertib, a selective ATP-competitive inhibitor of AKT, binds to its ATP-binding pocket (9), blocking AKT activation and subsequent downstream signaling, ultimately suppressing cancer cell growth and proliferation.

### 2.6. Other Targeted Therapies

Around 15–20% of CRPC tumors eventually become independent of AR signaling, though detecting this shift in clinical practice remains difficult; one notable sign is the histologic progression from AR-positive adenocarcinoma to AR-negative, poorly differentiated small cell neuroendocrine carcinoma [187]. Neuroendocrine prostate cancer (NEPC) represents a highly aggressive histological variant of PCa, typically emerging in advanced disease stages as a form of resistance to therapy. Its poor prognosis is largely due to delayed detection and the current absence of effective treatment options [188].

This tumor phenotype includes both pure small cell carcinomas and mixed adenocarcinoma–neuroendocrine morphologies [189]. While AR expression is generally low, even when present, NEPC tumors exhibit reduced reliance—or relative indifference—toward canonical AR signaling. Diagnosis is typically confirmed through a metastatic tumor biopsy revealing the characteristic morphology. NEPC is histologically distinct from prostate adenocarcinoma and is defined by small, round, blue neuroendocrine cells that typically lack AR expression and do not produce PSA. Instead, these cells commonly express neuroendocrine markers such as chromogranin A, synaptophysin, and neuron-specific enolase (NSE) [190]. Although standardized criteria for when to perform such a biopsy are lacking, it may be warranted in cases with unusually aggressive clinical behavior, atypical metastatic patterns, or disease progression accompanied by low or stable PSA levels. Current NCCN guidelines support considering metastatic biopsy in suspected NEPC transformation, as patients who develop small cell carcinoma may be eligible for platinum-based chemotherapy regimens, similar to those used in small cell lung cancer (SCLC) [189].

### 2.7. New Frontiers in PCa

#### Immunotherapy

PCa is frequently described as a “cold” tumor, characterized by a lack of tumor-infiltrating lymphocytes, low levels of neoantigen expression, and a high presence of immunosuppressive cells, all of which contribute to a tumor microenvironment that is poorly responsive to immune-based therapies [191]. To overcome these challenges, it is essential to gain a deeper understanding of the biological mechanisms involved and to develop more advanced combination treatments aimed at transforming “cold” tumors into ones that are more susceptible to immunotherapy [192]. Although immunotherapy has not yet shown major success in PCa, a deeper understanding of its immune-related barriers and the strategic use of drug combinations could be the key to achieving a cure [193].

Several approaches have been explored, including immune checkpoint inhibitors, enhancement of immunogenic cell death (ICD), reversal of the immunosuppressive tumor microenvironment (TME), cancer vaccines, CAR-T cell therapy, and methods to overcome physical barriers within the TME. Each strategy comes with its own advantages, limitations, and potential clinical applications.

Immunotherapy restores the host’s impaired immune system to recognize and attack cancer cells, which has precise targeting and long-lasting efficacy, especially for cancer types that are refractory to conventional therapies.

***Immune checkpoint inhibitors***

Immune checkpoint blockade is a key immunotherapy strategy targeting molecules like CTLA-4, PD-1, and PD-L1, which are involved in suppressing immune responses and allowing tumor cells to evade detection [194]. While effective in other cancers, checkpoint inhibitors have shown limited success in PCa, largely due to its immunologically “cold” tumor environment [195]. CTLA-4 regulates T cell activation in lymphoid tissues, while PD-1/PD-L1 interactions suppress T cell activity at the tumor site [196]. In PCa, PD-L1 expression is variable and higher in metastatic cases, but it does not reliably predict treatment response [197]. Monotherapy with drugs like ipilimumab (anti-CTLA-4) or PD-1/PD-L1 inhibitors (e.g., nivolumab, pembrolizumab) has produced modest results, with low objective response rates and limited impact on OS [198].

However, combination therapies, particularly nivolumab with ipilimumab, have shown improved outcomes in select patients, especially those with genetic alterations such as high tumor mutational burden (TMB), microsatellite instability (MSI-H), DNA mismatch repair deficiency (dMMR), or CDK12 mutations [199,200]. These biomarkers are rare but are associated with better responses to immune checkpoint inhibitors [200]. This trial named CHECKMATE650 included a total of 90 patients with mCRPC were divided into two groups based on treatment timing: a pre-chemotherapy cohort and a post-chemotherapy cohort. In cohort 1 (before chemotherapy), the ORR was 25%, with a median OS of 19.0 months. In cohort 2 (after chemotherapy), the ORR dropped to 10%, and the median OS was 15.2 months. Notably, four patients—two from each cohort—achieved a complete response. However, multiple grade 3–4 adverse events were observed, indicating that further optimization of dosage and scheduling is essential to improve the safety profile [200]. Beyond these headline results, a deeper analysis highlights the main challenges of dual checkpoint blockade in PCa. Median rPFS remained modest (5.5 and 3.8 months in pre- and post-chemotherapy cohorts, respectively), and PSA responses occurred in only 18% and 10% of patients, reflecting the overall limited disease control [201]. Importantly, treatment was associated with substantial toxicity, with grade 3–4 immune-related adverse events in 42–53% of patients and four treatment-related deaths, leading to early discontinuation in many cases [201]. Exploratory biomarker analyses showed that responses were enriched in patients with high TMB (ORR 50% vs. 5.3% in TMB-low), DNA damage repair (DDR) defects, or PD-L1 ≥ 1%. These findings support the notion that PCa is an immunologically “cold” tumor, where only selected subgroups benefit meaningfully from checkpoint inhibition. Overall, CheckMate 650 underscores both the promise and the limitations of nivolumab plus ipilimumab: while durable complete responses can occur, the majority of unselected patients derive limited benefit, highlighting the urgent need for biomarker-driven strategies and safer dosing regimens in mCRPC [200].

In the randomized, double-blind phase III KEYNOTE-921 trial (*n* ≈ 1 030) of Pembrolizumab plus Docetaxel versus docetaxel + placebo in patients with previously treated mCRPC, the addition of pembrolizumab did not produce a statistically significant improvement in radiographic progression-free survival (median rPFS 8.6 vs. 8.3 months; HR 0.85 or median OS 19.6 vs. 19.0 months; HR 0.92; *p* = 0.17). Grade ≥ 3 treatment-related adverse events were more frequent with the pembrolizumab arm (43.2% vs. 36.6%) [201].

These negative results further emphasize the limited role of immunotherapy in unselected PCa populations [201].

Taken together, while preclinical studies have generated enthusiasm for immune checkpoint blockade in PCa, clinical evidence remains modest. Both CheckMate 650 and KEYNOTE-921 demonstrate that, in unselected mCRPC populations, objective responses are low and survival benefits are not significant, with considerable toxicity in combination settings [200,201]. These outcomes reflect the unique immunobiology of PCa, including an immunosuppressive tumor microenvironment with few tumor-infiltrating lymphocytes, low mutational burden and neoantigen load, and intrinsic resistance mechanisms to checkpoint inhibition. Moreover, PD-L1 expression is inconsistent and not reliably predictive of response. This gap between strong mechanistic rationale and disappointing clinical efficacy underscores the urgent need for biomarker-driven patient selection and rational combination strategies to overcome the immunosuppressive landscape of PCa [200,201].

***Enhancement of cancer cell ICD***

Regulated cell death (RCD) is a genetically programmed process that controls how cells die and has traditionally been seen as immunologically neutral or non-stimulatory [202]. However, when dying cells trigger adaptive immunity and long-term immune memory in genetically identical hosts, the process is termed immunogenic cell death (ICD) [203]. Two key features of ICD—antigenicity and adjuvanticity—are critical in activating an effective adaptive immune response. Various treatments such as chemotherapy, ionizing radiation, and certain physical interventions can promote ICD by causing tumor cells to release damage-associated molecular patterns (DAMPs) and tumor-associated antigens (TAAs) [203]. This leads to the transformation of the tumor microenvironment (TME) from an “immune-cold” (unresponsive) state to an “immune-hot” (responsive) state, enhancing the immune system’s ability to attack tumors [203]. As a result, both primary and metastatic tumor growth can be suppressed, representing a promising approach to boost the effectiveness of cancer immunotherapy [203].

***Reversal of the immunosuppressive tumor microenvironment (TME)***

The tumor microenvironment (TME) of PCa is composed of various pro-tumorigenic elements, including cancer-associated fibroblasts (CAFs), mesenchymal stem cells (MSCs), immune cells, and extracellular matrix (ECM) components. CAFs, the dominant stromal cells, promote tumor growth and invasion through ECM remodeling and secretion of factors like ROS and tenascin-C, driven by TGFβ signaling from PCa cells [112]. MSCs contribute to angiogenesis and immune suppression by promoting the development of myeloid-derived suppressor cells (MDSCs) [204]. MDSCs, along with tumor-associated macrophages (TAMs), support immune evasion by inducing regulatory T cells (Tregs) and suppressing effector immune responses via cytokines and immune checkpoint molecules [205]. The long latency and low mutational burden of PCa facilitate the development of a highly immunosuppressive TME, posing a major barrier to effective immune-based therapies in metastatic settings.

In PCa, myeloid-derived suppressor cells (MDSCs)Cs are key mediators of immunosuppression within the TME, contributing to immune evasion and disease progression [206]. Divided into monocytic (M-MDSCs) and polymorphonuclear (PMN-MDSCs) subtypes, these cells inhibit both CD4+ and CD8+ T cell functions through metabolic depletion, production of ROS/RNS, and immune checkpoint expression [207]. PMN-MDSCs, which dominate in prostate tumor tissues, are particularly potent in suppressing cytotoxic responses and are recruited via tumor-derived cytokines like IL-8 and GM-CSF. MDSCs are also involved in the development of castration resistance, partly through IL-23 and feedback loops involving IDO and IL-8 [208]. Their accumulation, activation, and interaction with other suppressive cells establish a tolerogenic microenvironment that limits the effectiveness of immunotherapies in advanced PCa.

***Adoptive T cell therapy***

Dendritic cell-based vaccines, particularly sipuleucel-T, remain the only immunotherapeutic approach approved for mCRPC, offering a modest OS benefit of about four months despite minimal effects on PSA levels or tumor burden [209]. Subsequent efforts using monocyte-derived dendritic cells (MoDCs) have shown limited clinical efficacy, although they can elicit immune responses [209]. These findings underscore the importance of optimizing antigen presentation and delivery, and have paved the way for exploring next-generation platforms—such as nanoparticles and viral vectors—for enhancing vaccine effectiveness in combination strategies [209].

***Therapeutic cancer vaccines***

Over the past decades, numerous vaccine strategies have been investigated for PCa, including cell-based, microbial, peptide/protein, and nucleic acid-based platforms [210]. Among these, sipuleucel-T remains the only FDA-approved vaccine to demonstrate a survival benefit in patients with mCRPC, although its impact is modest and clinical use is limited by cost and complexity [210]. Other vaccines, such as PROSTVAC or GVAX, failed to show significant benefit in phase III trials, but these efforts have laid the groundwork for more advanced platforms like mRNA vaccines, currently under early investigation [210].

## 3. Conclusions

Metastatic prostate cancer remains a complex and heterogeneous disease, requiring a multimodal approach that integrates established standards of care with innovative therapeutic strategies. Recent advances, including next-generation androgen receptor inhibitors, targeted therapies such as PARP inhibitors, radioligand treatments, PI3K/AKT pathway inhibitors, and emerging immunotherapeutic approaches, are reshaping the treatment landscape and improving patient outcomes. Precision medicine, driven by molecular profiling, is becoming increasingly central to tailoring interventions and optimizing benefit–risk ratios. Nonetheless, significant challenges remain, including the development of resistance, the need for more effective options in aggressive variants, and the integration of novel agents into earlier disease settings. Future research should focus on well-designed clinical trials, combinatorial strategies, and translational studies aimed at deepening our understanding of tumor biology, ultimately paving the way toward more personalized and durable disease control.

## Figures and Tables

**Table 1:** Therapies for mHSPC: evidence from major clinical trials.

Therapy	mHSPC	Results Trial
ADT * + Second-generation of Androgen Synthesis Inhibitor (target CYP17)	**Abiraterone + Prednisone** *– LATITUDE, STAMPEDE*	– Both trials demonstrated improved OS and rPFS over ADT alone, particularly in high-risk or high-volume disease.
ADT * + Second generation of Androgen Receptor Inhibitor	**(1) Enzalutamide** – *ENZAMET*, *ARCHES* **(2) Apalutamide** – *TITAN*	(1)—Prolonged OS and rPFS; benefit consistent across disease volume and prior docetaxel use – Improved time to PSA progression (2)—Significant OS and rPFS benefit vs ADT alone – Manageable safety profile; consistent efficacy across subgroups
ADT * + Docetaxel + Abiraterone	**Triplet therapy** – *PEACE-1 trial*	– Improved OS and rPFS vs ADT + Docetaxel, especially in high-volume disease; no unexpected toxicities
ADT * + Docetaxel + Darolutamide	**Triplet therapy** – *ARASENS trial*	– Significant OS benefit vs ADT + Docetaxel; consistent efficacy across subgroups; safety similar to control

* **ADT** refers to therapeutic approach designed to suppress androgen production by interfering with the hypothalamic-pituitary-gonadal axis; under this definition are included both LHRH agonists and antagonists. Abbreviations: overall survival (OS); radiographic progression-free survival (rPFS).

**Table 2:** Chemotherapy in mCRPC: Evidence from major Clinical Trials.

Disease Setting	Treatment Regimen	Clinical Trials	Results Trial
*mCRPC (1st line)*	ADT * + Docetaxel	GETUG-AFU 15, CHAARTED	*CHAARTED* demonstrated a significant improvement in overall survival (OS), particularly in patients with high-volume disease. *GETUG-AFU 15* showed a similar trend but did not reach statistical significance for OS benefit.
*mCRPC (2nd line)*	ADT * + Cabazitaxel	TROPIC trial (Phase III)	*TROPIC* showed superior OS, progression-free survival (PFS), and PSA response rates compared with mitoxantrone in patients previously treated with docetaxel. Main toxicities included neutropenia and diarrhea, but overall treatment was manageable.
*mCRPC (3rd line)*	ADT * + Taxane + Platinum-based chemotherapy	Corn et al. (Phase I–II), RECARDO trial (Phase II)	Corn et al. and *RECARDO* trials indicated modest activity of taxane–platinum combinations in heavily pretreated mCRPC, with occasional durable responses in patients with aggressive or neuroendocrine features. Toxicity was acceptable with appropriate dose management.

* **ADT** refers to therapeutic approach designed to suppress androgen production by interfering with the hypothalamic-pituitary-gonadal axis; under this definition are included both LHRH agonists and antagonists.

**Table 3:** Comparative overview of adverse events associated with PARP inhibitors in mCRPC clinical trials.

PARP Inhibitor/Combination	Study	Most Common Adverse Events (All Grades)	Grade ≥ 3 Events
**Olapar** **ib + Abiraterone + Prednisone**	PROpel (Phase III)	Anemia (52.5% asymptomatic, 45.2% symptomatic), fatigue/asthenia (34–46%), nausea (29–35%), diarrhea (16–22%), decreased appetite (10–18%), vomiting (14–18%), hypertension (13–14%), neutropenia (8–14%), musculoskeletal pain	Anemia ≥ 3: 14–20%, overall grade ≥ 3 AEs: 54–58% (vs. 42–46% placebo)
**Niraparib + Abiraterone + Prednisone (AAP)**	MAGNITUDE (Phase III)	Fatigue, nausea, anemia, hypertension, GI toxicities; overall manageable safety profile	Main ≥ 3 events: anemia, hypertension (higher in HRR+ subgroup)
**Rucaparib (monotherapy)**	TRITON2 (Phase II)	Fatigue, nausea, decreased appetite, constipation	Anemia ≥ 3: ~25%
**Talazoparib + Enzalutamide**	TALAPRO-2 (Phase III)	Anemia, neutropenia, fatigue	Anemia most common grade 3–4 AE; neutropenia significant

## Data Availability

No new data were created or analyzed in this study. Data sharing is not applicable to this article.
